# ESHAP and G-CSF is a superior blood stem cell mobilizing regimen compared to cyclophosphamide 1.5 g m^−2^ and G-CSF for pre-treated lymphoma patients: a matched pairs analysis of 78 patients

**DOI:** 10.1054/bjoc.1999.0915

**Published:** 2000-01-18

**Authors:** M J Watts, S J Ings, D Leverett, A MacMillan, S Devereux, A H Goldstone, D C Linch

**Affiliations:** Department of Haematology, University College London, 98 Chenies Mews, London, WC1E 6HX, UK; Department of Haematology, Mount Vernon Hospital, Northwood, Middlesex HA6 2JR, UK

**Keywords:** lymphoma, PBSC, ESHAP, cyclophosphamide, mobilization

## Abstract

Cyclophosphamide 1.5 g m^−2^followed by granulocyte colony-stimulating factor (G-CSF) is an effective peripheral blood stem cell (PBSC) mobilizing regimen, but has limited anti-lymphoma activity. We therefore assessed the mobilizing potential of ESHAP (etoposide, ara-C, methylprednisolone and cisplatin), a potent second-line lymphoma regimen followed by G-CSF. The results were compared in 78 patients with relapsed or resistant lymphomas with the use of cyclophosphamide 1.5 g m^−2^followed by G-CSF in a matched pairs analysis, matching the ESHAP recipients (for predetermined prognostic factors) from a cohort of 178 lymphoma patients mobilized with cyclophosphamide and G-CSF. The total numbers of mononuclear cells collected at apheresis was similar with both regimens but ESHAP plus G-CSF resulted in a significantly higher percentage of CD34+ cells, absolute number of CD34+ cells and GM-CFC (all with *P* -values < 0.001). The number of patients requiring only one apheresis harvest to achieve a CD34+ cell yield of > 2.0 × 10^6^kg^−1^was greatly increased in the ESHAP recipients (56/78 vs 17/78, *P*< 0.001). The total number of progenitor cells collected was not significantly different with the two mobilization regimens because of this higher number of apheresis in the cyclophosphamide group. The proportion of patients who failed to achieve a minimum CD34+ cell target of 1 × 10^6^kg^−1^with the pooled harvests was less in the ESHAP arm (four patients vs nine patients) despite an increased number of aphereses in the cyclophosphamide recipients. ESHAP plus G-CSF is well tolerated and is an excellent mobilization regimen in patients with pre treated lymphoma. © 2000 Cancer Research Campaign


*British Journal of Cancer* (2000) **82**(2), 278–282
© 2000 Cancer Research Campaign
Article no. bjoc.1999.0915

### ESHAP and G-CSF is a superior blood stem cell mobilizing regimen compared to cyclophosphamide 1.5 g m$^{-2}$ and G-CSF for pre-treated lymphoma patients: a matched pairs analysis of 78 patients

**MJ Watts[1], SJ Ings[1], D Leverett[1], A MacMillan[2], S Devereux[1], AH Goldstone[1] and DC Linch[1]**

[1]Department of Haematology, 98 Chenies Mews, University College London, London WC1E 6HX, UK, [2]Department of Haematology, Mount Vernon Hospital, Northwood, Middlesex HA6 2JR, UK

**Summary** Cyclophosphamide 1.5 g m$^{-2}$ followed by granulocyte colony-stimulating factor (G-CSF) is an effective peripheral blood stem cell (PBSC) mobilizing regimen, but has limited anti-lymphoma activity. We therefore assessed the mobilizing potential of ESHAP (etoposide, ara-C, methylprednisolone and cisplatin), a potent second-line lymphoma regimen followed by G-CSF. The results were compared in 78 patients with relapsed or resistant lymphomas with the use of cyclophosphamide 1.5 g m$^{-2}$ followed by G-CSF in a matched pairs analysis, matching the ESHAP recipients (for predetermined prognostic factors) from a cohort of 178 lymphoma patients mobilized with cyclophosphamide and G-CSF. The total numbers of mononuclear cells collected at apheresis was similar with both regimens but ESHAP plus G-CSF resulted in a significantly higher percentage of CD34+ cells, absolute number of CD34+ cells and GM-CFC (all with *P*-values < 0.001). The number of patients requiring only one apheresis harvest to achieve a CD34+ cell yield of > $2.0 \times 10^6$ kg$^{-1}$ was greatly increased in the ESHAP recipients (56/78 vs 17/78, $P < 0.001$). The total number of progenitor cells collected was not significantly different with the two mobilization regimens because of this higher number of apheresis in the cyclophosphamide group. The proportion of patients who failed to achieve a minimum CD34+ cell target of $1 \times 10^6$ kg$^{-1}$ with the pooled harvests was less in the ESHAP arm (four patients vs nine patients) despite an increased number of aphereses in the cyclophosphamide recipients. ESHAP plus G-CSF is well tolerated and is an excellent mobilization regimen in patients with pre treated lymphoma. © 2000 Cancer Research Campaign

**Keywords**: lymphoma; PBSC; ESHAP; cyclophosphamide; mobilization

Haemopoietic stem/progenitor cells can be mobilized into the peripheral blood using granulocyte colony stimulating factor (G-CSF) and granulocyte-macrophage CSF (GM-CSF), either alone or following chemotherapy (Watts and Linch, 1997). Many different mobilizing chemotherapy regimens have been employed, with single-agent cyclophosphamide one of the most frequent. The doses of cyclophosphamide used have varied between 1 g m$^{-2}$ and 7 g m$^{-2}$ and at our institution we have used 1.5 g m$^{-2}$ (Jones et al, 1994; Watts et al, 1997*b*, 1998). This dose of cyclophosphamide followed by G-CSF is an effective mobilizing regimen in that the minimum required number of CD34+ cells could be collected in two aphereses in 90% of patients with previously treated lymphoma (Watts et al, 1997*b*). A major advantage of this regimen is that it can be given as an out-patient, few patients (5%) require admission for the treatment of chemotherapy-related complications and the stem/progenitor cell mobilization kinetics are highly predictable (Watts et al, 1995, 1997*b*).

There is some evidence that higher doses of cyclophosphamide result in greater progenitor/stem cell mobilization (Rowlings et al, 1992; Goldschmidt et al, 1996; Schwartzberg et al, 1998) but the complication rate of the procedure rises dramatically. One study suggested that combination chemotherapy was superior to intermediate dose cyclophosphamide (McQuaker et al, 1997) but another comparing cyclophosphamide 4.5 g m$^{-2}$ with a combination of cyclophosphamide and etoposide found no advantage to the more toxic combination therapy (Ketterer et al, 1997).

Single-agent cyclophosphamide at a dose of 1.5 g m$^{-2}$ is not, however, an optimal anti-lymphoma regimen particularly in patients who have just failed a cyclophosphamide-containing combination chemotherapy regimen. We have therefore explored the use of ESHAP (etoposide, ara-C, methylprednisolone and cisplatin) as a mobilizing regimen (Watts et al, 1996) as it is a proven lymphoma salvage regimen and contains no highly stem cell-toxic alkylating agents which might mitigate against effective mobilization (Velasquez et al, 1994).

We report here ESHAP/G-CSF mobilization of 84 patients with lymphoma (Hodgkin's disease, low-grade and high-grade non-Hodgkin's lymphoma (NHL)). To allow meaningful comparison with the results obtained with cyclophosphamide 1.5 g m$^{-2}$ we have carried out matched pairs analysis with cyclophosphamide-mobilized patients, matching for those factors that can influence mobilization efficacy.

### PATIENTS STUDIED

Eighty-four patients with lymphoma have received ESHAP chemotherapy followed by G-CSF (as detailed below) prior to collection of peripheral blood stem cell (PBSC) at UCLH since October 1995. Matching was carried out using a database

*Received 3 March 1999*
*Revised 6 July 1999*
*Accepted 2 August 1999*

*Correspondence to:* DC Linch containing 178 lymphoma patients mobilized with cyclophosphamide 1.5 g $m^{-2}$ between July 1992 and October 1997. The latter patients comprised 71 with Hodgkin's disease, 50 with low-grade NHL and 63 with high-grade NHL. The two groups were of similar weights, the median value being 75 kg in the ESHAP group (range 43–126 kg) and 73.5 kg in the cyclophosphamide group (range 47–103 kg).

### Matching criteria

Successful matches were determined using criteria which we have previously shown to influence mobilization in a cohort of lymphoma patients at our centre (Watts et al, 1997*b*). These included matching for diagnosis, receipt of previous radiotherapy and mini-BEAM therapy. Having fulfilled these criteria the cyclophosphamide mobilized patient was then selected on the basis of the number of chemotherapy cycles the patient had received with a limit of only ± 2 cycles allowed. No patient in this series had microscopic evidence of bone marrow involvement at the time of mobilization. All of these factors have been demonstrated in a number of studies to affect progenitor yields (Haas et al, 1994; Bensinger et al, 1995; Morton et al, 1997; Weaver et al, 1998).

### Mobilization regimens and apheresis

The patients mobilized with low-dose cyclophosphamide (1.5 g $m^{-2}$) were given this drug intravenously (i.v.) on day 1, followed by G-CSF given subcutaneously (s.c.) at 10 μg $kg^{-1}$ (filgrastim) or a single vial of lenograstim (263 μg) 24 h afterwards, and daily thereafter until harvesting was complete. Apheresis commenced on a rising WBC from the neutropenic nadir, the optimal first harvest progenitor yields were obtained when the WBC first exceeded $5.0 \times 10^9 l^{-1}$ (Watts et al, 1995) typically on day 10 (range 8–12). In 77/78 of the cyclophosphamide group this WBC was achieved at first harvest. In one patient the recovery WBC only attained $3.2 \times 10^9 l^{-1}$ by day 14 when apheresis commenced (Table 1). The ESHAP protocol (Velasquez et al, 1994) involved overnight hydration followed by etoposide at 40 mg $m^{-2}$ i.v. days 1–4, cisplatin at 25 mg $m^{-2}$ days 1–4, cytarabine 2 g $m^{-2}$ day 1 and methyl-prednisolone 500 mg i.v. days 1–5. This was followed on day 6 with daily G-CSF as for the cyclophosphamide-mobilized patients until completion of harvest. The first harvest collected with this protocol was on day 15 providing the recovery WBC exceeded $3.0 \times 10^9 l^{-1}$ (range day 15–18). The WBC kinetics of the ESHAP mobilization protocol were established with frequent blood counts in the early part of the study (Figure 1).

**Table 1** Clinical factors matched between ESHAP + G-CSF and cyclophosphamide + G-CSF mobilized patients

| Diagnosis | (*n*) | Prior RT | Prior mini-BEAM | Prior RT *and* mini-BEAM | Prior cycles of chemo[a] |
|---|---|---|---|---|---|
| HD | 26 | 14/26 | 6/26 | 2/26 | 8 (2–16) |
| HGNHL | 41 | 6/41 | 5/26 | 0/26 | 7 (3–14) |
| LGNHL | 11 | 1/11 | 1/11 | 0/11 | 8 (5–17) |

[a]One month continuous alkylating therapy counted as one cycle of chemotherapy.

All of the final 78 clinically matched ESHAP/G-CSF mobilized patients were harvested on a continuous apheresis machine. Sixty-five patients were collected on a Baxter CS3000 (Baxter Healthcare Ltd, Berkshire, UK) set to process a fixed 10 l blood volume and the remaining 13 patients collected on a COBE Spectra (COBE Laboratories Ltd, Gloucester, UK.) with a median of 11.8 l processed. Sixty-six of the cyclophosphamide/G-CSF-matched patients were also harvested on these machines, 47 on the Baxter machine as described and 19 on the COBE machine (median 12.2 l blood volume processed). Twelve patients in the cyclophosphamide-mobilized group were harvested with an intermittent collection device, the Haemonetics V50 (Haemonetics Ltd, Leeds, UK) as previously described (Jones et al, 1994; Watts et al, 1997*b*). The progenitor yield comparison between the two mobilization protocols in the present study was performed with and without the 12 patients pairs which included intermittent apheresis technology. One to three apheresis harvests were collected, the number of aphereses being determined by the progenitor yields obtained.

### Progenitor cell assays

A sterile sample from each harvest was diluted 1/10 (for cell counts) and 1/100 (for colony assays) in RPMI containing 10% fetal calf serum and 20 U $ml^{-1}$ heparin. Harvest cell counts, CD34-positive cell numbers and granulocyte/monocyte-colony forming cells (GM-CFC) were performed as described previously (Watts et al, 1997*b*).

### RESULTS

### Toxicity of ESHAP regimen

In all 84 patients, ESHAP was administered as in-patient therapy with discharge following the cisplatin infusion. Seven patients (8%) required subsequent readmission to hospital prior to their apheresis date, four with fevers and presumed sepsis, three of whom had severe neutropenia ($< 0.5 \times 10^9 l^{-1}$) and one who was never neutropenic. Two patients were admitted for platelet transfusions (platelets $< 15 \times 10^9 l^{-1}$) although neither was bleeding or septic. A further patient was admitted with chest pain for exclusion of a pulmonary embolus. His blood counts were in the normal range and no cause of the chest pain was ever discovered.

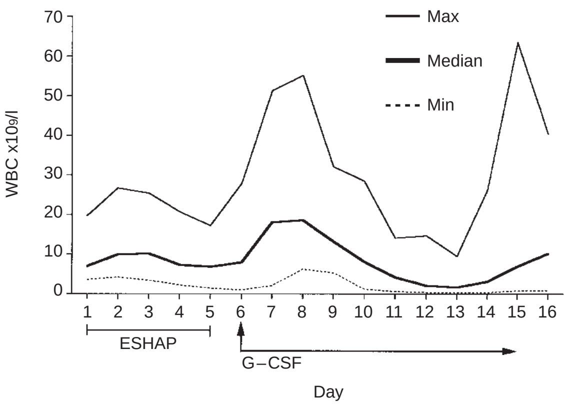

**Figure 1** WBC kinetics following mobilization with ESHAP and G-CSF

**Table 2** Disease status at the time of PBSC collection

| Status at mobilization | Cyclophosphamide-mobilized patients | ESHAP-mobilized patients |
|---|---|---|
| At diagnosis | 1 | 0 |
| PR/CR to first-line therapy | 22 | 24 |
| Primary refractory to first line therapy | 15 | 11 |
| First relapse | 23 | 28 |
| Beyond first relapse | 17 | 15[a] |
| Total | 78 | 78 |

[a]Six patients had received prior stem cell transplants.

### Matching of ESHAP recipients to cyclophosphamide 1.5 g $m^{-2}$ recipients

Seventy-eight of the 84 ESHAP recipients could be matched for criteria likely to influence mobilization efficiency as detailed in the Methods section. The frequency of these factors for each histological type, and the number of prior chemotherapy cycles is detailed in Table 2. Furthermore, when the matched ESHAP patients were compared to the cyclophosphamide recipients there was no difference in sex, age, receipt of prior alkylating therapy and time from last chemotherapy to mobilization chemotherapy. The disease status of the two mobilization groups at the time of mobilization is summarized in Table 1.

### Comparison of harvest yields for matched ESHAP and cyclophosphamide recipients

The mobilization results for the matched pairs are shown in Table 3. Data is shown for the first harvest and for the total of all harvests collected. The number of MNC was similar with both groups but the number of CD34+ cells and GM-CFC was significantly greater ($P$ < 0.001 for both). When total yields were compared the result with ESHAP was again significantly better, although the differences were less marked than for the first harvest. This is because 56 (72%) of ESHAP recipients only had one harvest (all with more than $2 \times 10^6$ $kg^{-1}$ CD34+ cells from a single harvest), whereas only 17 (22%) of cyclophosphamide recipients had only one harvest. The average number of collections was 1.4 for ESHAP recipients and 2.1 for cyclophosphamide recipients.

Twelve of the matched cyclophosphamide recipients had been apheresed using an intermittent flow machine, whereas all the ESHAP recipients had been apheresed with a continuous flow device. To ensure that the use of the intermittent flow device had not prejudiced the results in the cyclophosphamide patients, the comparative analysis was repeated considering only the matched pairs who had been collected on a continuous flow machine. The results were very similar to when all 78 pairs were considered. For instance, the median number of CD34+ cells collected with the first harvest was $4.1 \times 10^6$ $kg^{-1}$ in the ESHAP recipients and $1.9 \times 10^6$ $kg^{-1}$ in the cyclophosphamide recipients ($P$ = 0.006). The corresponding values for GM-CFC were $4.8 \times 10^5$ $kg^{-1}$ and $2.7 \times 10^5$ $kg^{-1}$ respectively ($P$ < 0.001).

In practice the median number of progenitor cells collected is less important than the proportion of patients achieving predefined threshold levels. The proportion of patients achieving the various thresholds is shown in Table 4.

The six ESHAP recipients who could not be matched were all successfully mobilized, with four out of the six having over $1 \times 10^6$ $kg^{-1}$ CD34+ cells in the first collection the median value being $5.7 \times 10^6$ $kg^{-1}$. Exclusion of these patients did not therefore significantly influence the results obtained.

### Haematological recovery

Sixty-five of the cyclophosphamide mobilized patients and 60 of the ESHAP mobilized patients are evaluable for engraftment. The median time to engraftment was similar for both groups (12 days and 11 days to a neutrophil count of > $0.5 \times 10^9$ $l^{-1}$ and 11 days and 12 days to platelet independence, defined as an unsupported platelet count above $15 \times 10^9$ $l^{-1}$) in the cyclophosphamide and ESHAP groups respectively. There were ten patients with slow (>21 days) platelet recovery in the cyclophosphamide group compared to only five in the ESHAP group but this difference was not significant.

**Table 3** Apheresis characteristics and progenitor yields obtained at first harvest and in total apheresis collections in 78 ESHAP+ G-CSF-mobilized patients compared to matched cyclophosphamide + G-CSF-mobilized patients

| | ESHAP + G-CSF | Cyclophosphamide + G-CSF | *P*-values |
|---|---|---|---|
| *First harvest yields* | | | (paired *t*-test) |
| PB WBC $\times 10^9$ $l^{-1}$ | 9.0 (2.9–63.6) | 10.0 (3.2[a]–51.9) | |
| Collection day | 15 (14–19) | 10 (8–16) | |
| MNC $\times 10^8$ $kg^{-1}$ | 2.0 (0.5–7.9) | 1.9 (0.7–7.5) | NS |
| CD34% | 2.4 (<0.1–20.1) | 0.9 (0.1–9.2) | <0.001 |
| CD34 $\times 10^6$ $kg^{-1}$ | 4.8 (<0.1–80.2) | 1.7 (0.1–28.8) | <0.001 |
| GM-CFC $\times 10^5$ $kg^{-1}$ | 4.9 (<0.1–86.0) | 2.3 (<0.1–11.8) | <0.001 |
| *Total harvest yields* | | | |
| Number of patients who had (1, 2, 3 or 4) apheresis collections performed respectively | (56, 15, 6, 1) | (17, 37, 24, 0) | |
| MNC $\times 10^8$ $kg^{-1}$ | 2.7 (0.5–9.2) | 3.7 (0.9–15.6) | 0.002 |
| CD34 $\times 10^6$ $kg^{-1}$ | 4.9 (<0.1–80.2) | 3.3 (0.2–41.0) | 0.032 |
| GM-CFC $\times 10^5$ $kg^{-1}$ | 6.1 (<0.1–86.0) | 4.3 (0.2–21.2) | 0.008 |

[a]In one patient the recovery WBC was particularly slow and was only $3.2 \times 10^9$/l on day 14 when apheresis commenced.

 © 2000 Cancer Research Campaign

**Table 4** Proportion of patients who failed to achieve various CD3+ cell threshold levels* or in the total harvest collected

| | Moilization group | CD34+ cell thresholds $\times 10^6$/kg | | |
|---|---|---|---|---|
| | | <1 | <2 | <3.5 |
| First harvest | ESHAP | 13 (16%) | 20 (26%) | 47 (60%) |
| | Cyclo | 24 (31%) | 43 (55%) | 19 (24%) |
| Total collected | ESHAP | 4 (5%) | 12 (15%) | 52 (67%) |
| | Cyclo | 9(12%) | 23 (29%) | 37 (47%) |

*The minimum yield to proceed to high dose therapy in our centre is $1\times10^6$/kg CD34+ cells and the aim is to collect $2\times10^6$/kg CD34+ cells were obtained additional aphereses were performed providing that the peripheral blood CD34+ cell count exceeded 10×10/L. The ideal yield is $3.5\times10^6$/kg CD34+ cells above which delayed platelet recovery is very infrequent (Watts et al, 1998).

### DISCUSSION

A large number of regimens have been used for stem cell mobilization. In some circumstances G-CSF alone is required (e.g. normal donors) or is adequate, but the general consensus is that improved yields are obtained with the combination of chemotherapy and growth factors. In many situations, such as relapsed and resistant lymphoma, the mobilizing protocol must also have good anti-tumour activity to test tumour chemosensitivity and effect bulk reduction.

Intermediate dose cyclophosphamide (1.5 g $m^{-2}$) plus G-CSF has been extensively used in our centre. It has the advantage of being able to be given as a day case, only causes complications requiring readmission in about 5% of cases, and is efficacious in the large majority of patients (Watts et al, 1997*b*, 1998). Some patients do fail to mobilize the required or desired number of progenitor cells, however, and single-agent cyclophosphamide at this dose is not optimal anti-lymphoma therapy. When greater anti-lymphoma activity has been required we have used the highly effective mini-BEAM or dexa-BEAM regimens, but their use is limited by stem cell toxicity with a reduction in quantity and quality of subsequent harvest yields (Dreger et al, 1995; Watts et al, 1997*b*; Weaver et al, 1998). We therefore chose to explore the value of the ESHAP regimen which is highly effective in a range of lymphoma types, is less toxic than the DHAP regimen, the forerunner to ESHAP, and contains no stem cell toxic agents (Velasquez et al, 1994). ESHAP was very well tolerated. Only seven out of 84 patients (8%) required re-admission following the administration of ESHAP which does not differ significantly from the re-admission rate following cyclophosphamide 1.5 g $m^{-2}$. Only two patients required platelet transfusions and when ESHAP is followed by G-CSF administration, as was given here for mobilization, severe protracted neutropenia was infrequent (Figure 1). In this study ESHAP was administered on an in-patient basis over 5 days, which is a clear disadvantage to the regimen. However, in selected patients ESHAP can be given on an out-patient basis which reduces the costs of the procedure.

ESHAP was found to be a highly effective mobilization regimen especially when it is considered that many of the patients in this series were heavily pretreated. The median yield of CD34+ cells with the first apheresis was $4.8 \times 10^6$ $kg^{-1}$ and in 84% of patients a threshold value of $2 \times 10^6$ $kg^{-1}$ was achieved with the first apheresis. These values are superior to our reported experience with cyclophosphamide 1.5 g $m^{-2}$ but considerable care must be exercised in interpreting such results in the absence of a suitable control group. Ideally, randomized controlled trials are required but this was not possible in this situation where many patients with poor prognosis disease were considered to require more potent anti-lymphoma therapy than could be achieved with intermediate dose cyclophosphamide alone. For this reason we have performed a match pairs analysis, matching for all the variables we have previously found to influence progenitor yield, made possible by the fact that we had previously mobilized 178 patients with the cyclophosphamide and G-CSF regimen. This analysis confirms the superiority of ESHAP + G-CSF as a mobilization regimen.

It should be noted that the higher CD34+ cell yield was achieved with a comparable MNC harvest so that the proportion of CD34+ cells in the harvest was highly significantly increased. This is likely to be due to the lympholytic effect of the high-dose steroids within the ESHAP regimen. The higher percentage of CD34+ cells in the harvest may be advantageous if CD34+ cell purification is being considered as we have previously shown that low CD34+ cell percentage is associated with lower final purities after clinical scale purification procedures (Watts et al, 1997*a*).

Both cohorts of patients had similar engraftment times but this relates to the fact that minimal progenitor thresholds were applied. It does indicate, however, that the quality of the ESHAP-mobilized cells is satisfactory. The cyclophosphamide patients required more aphereses. A total of 108 collections were performed in the ESHAP-mobilized patients compared to 163 in the cyclophosphamide-mobilized patients, and this has relevance to any cost–benefit comparison of the two mobilization regimens. Even taking this into account, ESHAP, which was generally given as an in-patient regimen is likely to be more expensive than cyclophosphamide. Its major benefit relates to the proven anti-lymphoma activity of ESHAP (Velasquez et al, 1994) and in patients who are in complete response at the time of mobilization and have no poor risk factors for mobilization cyclophosphamide remains a suitable mobilization regimen.

It is difficult to compare the ESHAP regimen with other combination chemotherapy-mobilizing regimens because of the different patient groups included in different series. In addition highly variable numbers of apheresis procedures have been performed and the results with the first apheresis are often not reported. Schwartzberg and colleagues compared two cyclophosphamide plus etoposide regimens (Schwartzberg et al, 1998) and achieved excellent CD34+ cell yields with both. The patient group consisted, however, of newly diagnosed patients with breast cancer who had only received prior adjuvant chemotherapy. McQuaker and colleagues (McQuaker et al, 1997) have reported results with the IVE (ifosphamide 9 g $m^{-2}$, VP16 600 mg $m^{-2}$ and etoposide 50 mg $m^{-2}$) regimen in a group of lymphoma patients more analogous to those in this series. Good mobilization was achieved with a median yield of $1.94 \times 10^6$ $kg^{-1}$ CD34+ cells per leukapheresis. This is apparently less than with ESHAP but it should be noted that by reporting the median per apheresis rather than for the first apheresis this will underestimate the efficiency of the regimen. The IVE regimen is likely to be more toxic than ESHAP (Zinzani et al, 1994) and the high dose of ifosphamide poses a risk of encephalitis which may make out-patient administration difficult. Whether such toxicity is acceptable will depend on the response rate compared to ESHAP, the proportion of patients proceeding to high-dose therapy and the long-term outcome of these patients. Randomized comparative trials are now required.

### REFERENCES

Bensinger W, Appelbaum F, Rowley S, Storb R, Sanders J, Lilleby K, Gooley T, Demirer T, Schiffman K, Weaver C & et al (1995) Factors that influence collection and engraftment of autologous peripheral-blood stem cells. *J Clin Oncol* **13**: 2547–2555

Dreger P, Kloss M, Petersen B, Haferlach T, Loffler H, Loeffler M and Schmitz N (1995) Autologous progenitor cell transplantation: prior exposure to stem cell-toxic drugs determines yield and engraftment of peripheral blood progenitor cell but not of bone marrow grafts. *Blood* **86**: 3970–3978

Goldschmidt H, Hegenbart U, Haas R and Hunstein W (1996) Mobilization of peripheral blood progenitor cells with high-dose cyclophosphamide (4 or 7 g $m^2$) and granulocyte colony-stimulating factor in patients with multiple myeloma. *Bone Marrow Transplant* **17**: 691–697

Haas R, Mohle R, Fruhauf S, Goldschmidt H, Witt B, Flentje M, Wannenmacher M and Hunstein W (1994) Patient characteristics associated with successful mobilizing and autografting of peripheral blood progenitor cells in malignant lymphoma. *Blood* **83**: 3787–3794

Jones HM, Jones SA, Watts MJ, Khwaja A, Mills W, Fielding, A, Goldstone AH and Linch DC (1994) Development of a simplified single-apheresis approach for peripheral-blood progenitor-cell transplantation in previously treated patients with lymphoma. *J- Clin- Oncol* **12**: 1693–1702

Ketterer N, Salles G, Tremisi P, Espinous D, Raba M, Dumontet C, Moullet I, Niedhardt-Berard E-M, Boufafia F and Coiffier B (1997) Analysis of factors influencing 300 peripheral blood progenitor collections in 273 patients treated for lymphoproliferative diseases. *Blood* **90**: 213a [abstract 939].

McQuaker IG, Haynes AP, Stainer C, Anderson S and Russell NH (1997) Stem cell mobilization in resistant or relapsed lymphoma: superior yield of progenitor cells following a salvage regimen comprising ifosphamide, etoposide and epirubicin compared to intermediate-dose cyclophosphamide [see comments]. *Br J Haematol* **98**: 228–233

Morton J, Morton A, Bird R, Hutchins C and Durrant S (1997) Predictors for optimal mobilization and subsequent engraftment of peripheral blood progenitor cells following intermediate dose cyclophosphamide and G-CSF. *Leuk Res* **21**: 21–27

Rowlings PA, Bayly JL, Rawling CM, Juttner CA and To LB (1992) A comparison of peripheral blood stem cell mobilisation after chemotherapy with cyclophosphamide as a single agent in doses of 4 g $m^{-2}$ or 7 g $m^{-2}$ in patients with advanced cancer. *Aust N Z J Med* **22**: 660–664

Schwartzberg LS, Weaver CH, Birch R, Manner C, Tauer K, Beeker T, Morgan-Ihrig C, MacAneny B, Leff R, Smith R, Hainsworth J, Greco T, Schwerkoske J, Murphy MN and Buckner CD (1998) A randomized trial of two doses of cyclophosphamide with etoposide and G-CSF for mobilization of peripheral blood stem cells in 318 patients with stage II–III breast cancer. *J Hematother* **7**: 141–150

Velasquez WS, McLaughlin P, Tucker S, Hagemeister FB, Swan F, Rodriguez MA, Romaguera J, Rubenstein E and Cabanillas F (1994) ESHAP–an effective chemotherapy regimen in refractory and relapsing lymphoma: a 4-year follow-up study [see comments]. *J Clin Oncol* **12**: 1169–1176

Watts MJ and Linch DC (1997) Peripheral blood stem cell transplantation. *Vox Sang* **73**: 135–142

Watts MJ, Sullivan AM, Jaimeson E, Chavda N, Fielding A, Goldstone AH and Linch DC (1995) A single apheresis algorithm for PBSCT. *Br J Haematol* **89**: 22a (abstract 79).

Watts MJ, Leverett D, Sullivan AM, Peniket AJ, Perry AR, MacMillan A, Goldstone AH and Linch DC (1996) A comparison of ESHAP+G-CSF versus cyclophosphamide 1.5 $g/m^2$+G-CSF for PBSC mobilisation in pretreated lymphoma patients. *Blood* **88**: 396a

Watts MJ, Sullivan AM, Ings SJ, Leverett D, Peniket AJ, Perry AR, Williams CD, Devereux S, Goldstone AH and Linch DC (1997*a*) Evaluation of clinical scale CD34+ cell purification: experience of 71 immunoaffinity column procedures. *Bone Marrow Transplant* **20**: 157–162

Watts MJ, Sullivan AM, Jamieson E, Pearce R, Fielding A, Devereux S, Goldstone AH and Linch DC (1997*b*). Progenitor-cell mobilization after low-dose cyclophosphamide and granulocyte colony-stimulating factor: an analysis of progenitor-cell quantity and quality and factors predicting for these parameters in 101 pretreated patients with malignant lymphoma. *J Clin Oncol* **15:** 535–546

Watts MJ, Sullivan AM, Leverett D, Peniket AJ, Perry AR, Williams CD, Devereux S, Goldstone AH and Linch DC (1998) Back-up bone marrow is frequently ineffective in patients with poor peripheral-blood stem-cell mobilization. *J Clin Oncol* **16**: 1554–1560

Weaver CH, Zhen B and Buckner CD (1998) Treatment of patients with malignant lymphoma with Mini-BEAM reduces the yield of CD34+ peripheral blood stem cells [letter]. *Bone Marrow Transplant* **21**: 1169–1170

Zinzani PL, Barbieri E, Visani G, Gherlinzoni F, Perini F, Neri S, Bendandi M, Ammendolia I, Salvucci M, Babini L & et al (1994) Ifosfamide, epirubicin and etoposide (IEV) therapy in relapsed and refractory high-grade non-Hodgkin's lymphoma and Hodgkin's disease. *Haematologica* **79**: 508–512

 *© 2000 Cancer Research Campaign*
